# The CD11a partner in *Sus scrofa *lymphocyte function-associated antigen-1 (LFA-1): mRNA cloning, structure analysis and comparison with mammalian homologues

**DOI:** 10.1186/1746-6148-1-5

**Published:** 2005-10-10

**Authors:** Philippe GAC Vanden Bergh, Thomas Fett, Laurent LM Zecchinon, Anne VT Thomas, Daniel JM Desmecht

**Affiliations:** 1 Pathology Department, Faculty of Veterinary Medicine, University of Liege, Colonster Boulevard 20 B43, B-4000 Liege, Belgium

## Abstract

**Background:**

Lymphocyte function-associated antigen-1 (LFA-1, CD11a/CD18, alphaLbeta2), the most abundant and widely expressed beta2-integrin, is required for many cellular adhesive interactions during the immune response. Many studies have shown that LFA-1 is centrally involved in the pathogenesis of several diseases caused by Repeats-in-toxin (RTX) -producing bacteria.

**Results:**

The porcine-LFA-1 CD11a (alpha) subunit coding sequence was cloned, sequenced and compared with the available mammalian homologues in this study. Despite some focal differences, it shares all the main characteristics of these latter. Interestingly, as in sheep and humans, an allelic variant with a triplet insertion resulting in an additional Gln-744 was consistently identified, which suggests an allelic polymorphism that might be biologically relevant.

**Conclusion:**

Together with the pig CD18-encoding cDNA, which has been available for a long time, the sequence data provided here will allow the successful expression of porcine CD11a, thus giving the first opportunity to express the *Sus scrofa *beta2-integrin LFA-1 *in vitro *as a tool to examine the specificities of inflammation in the porcine species.

## Background

Integrins constitute a large family of adhesion molecules with important roles in cell-extracellular matrix and cell-cell interactions which condition both the maintenance of tissue integrity and the promotion of cellular migration. They are heterodimeric membrane glycoproteins composed of non-covalently associated single-pass transmembrane α and β subunits, which are expressed on a wide range of cells [1]. The biggest part of each integrin subunit is extracellular while transmembrane region and cytoplasmic tail are typically reduced. The N-terminal domains of the α and β subunits associate to form the integrin headpiece, which contains the ligand binding site. The C-terminal segments traverse the plasma membrane and mediate interactions with the cytoskeleton and with signalling proteins [2,3].

Among the integrins, the leukocyte-specific β_2_-integrins (CD11/CD18) include four members: (i) CD11a/CD18 (LFA-1, α_L_β_2_) on all leukocytes ; (ii) CD11b/CD18 (Mac-1, CR3, α_M_β_2_) mainly on myeloid cells ; (iii) CD11c/CD18 (gp150/95, CR4, α_X_β_2_, Leu-M5) and (iv) CD11d/CD18 (α_D_β_2_) on monocytes and macrophages [4]. The individuals lacking functional β_2 _integrins due to mutations in the β_2 _(CD18) subunit develop the LAD (lymphocyte adhesion deficiency) I syndrome characterized by repeated infections. This disease demonstrated that β_2 _integrins are of relevant importance in (i) leukocyte development and maturation, (ii) naïve cells circulation in secondary lymphoid tissues and (iii) leukocytes transendothelial migration to injured tissue [5-7].

Lymphocyte function-associated antigen-1 (LFA-1, CD11a/CD18, α_L_β_2_), the most abundant and widespread in expression β_2_-integrin, binds to the membrane proteins termed intercellular adhesion molecules ICAM-1 to ICAM-5 [4,8-12]. Several studies have shown that LFA-1 is centrally involved in the pathogenesis of diseases caused by Repeats-in-toxin (RTX) -producing bacteria. The virulence of both *Actinobacillus actinomycetemcomitans *(stomatitis in humans) and *Mannheimia haemolytica *(pneumonia in cattle) is clearly associated with the ligand-receptor interactions between their respective leukotoxin and CD11a/CD18, which triggers the synthesis and release of a wide array of cytokines and chemoattractants that exacerbate inflammation, and ultimately results in massive leukolysis [13,14]. As *Actinobacillus pleuropneumoniae*, the main causative agent of pneumonia in pigs, also produces toxins of the RTX family (*Apx *toxins) [15], it is tempting to hypothesize that the pathogenesis of the disease similarly rely on an interaction with the porcine LFA-1. On a more practical point of view, increasing our knowledge about this putative interaction could help the pig industry in controlling the economical losses and antibiotics abuses that are currently associated with *A. pleuropneumoniae *pneumonia [16]. The *Sus scrofa *CD18 (β_2_-) subunit has been well characterized [17], which is not the case of its partner in the LFA-1 heterodimer, CD11a. The purpose of this study is to report the cloning, sequencing and analysis of a cDNA encoding porcine CD11a, thus giving the first opportunity to produce recombinant LFA-1 for studies focused on interactions between Apx toxins and swine LFA-1.

## Results and discussion

### Characterization of PoCD11a-encoding cDNA and deduced amino-acid sequence

**Figure 1:**
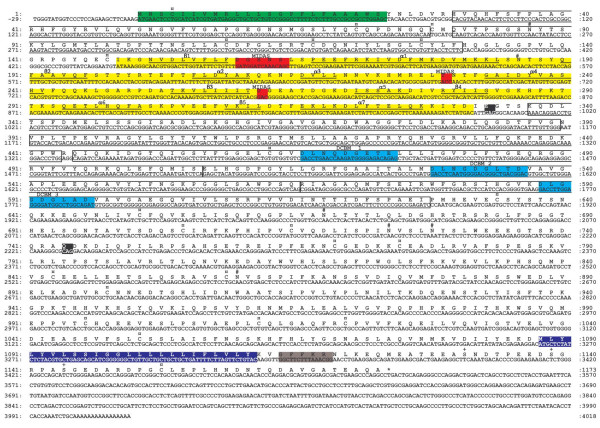
**The nucleotide and deduced amino acid sequences of *Sus scrofa *CD11a cDNA**. The putative leader peptide and transmembrane region are respectively represented by green and dark blue boxes. The αI-domain is showed by a yellow box. Its five-stranded β-sheets and seven α-helices are underlined. Its metal-ion dependent adhesion site (MIDAS) is represented by red boxes. The sequences of the seven repeats that surround the αI-domain are framed. Light blue boxes represent the central divalent cation-binding motifs (DCBM) of the three COOH-terminal repeats. The important Glu-333 residue (E) and the supplementary Gln-744 (Q) are in black boxes. The conserved sequence "GFFKR" of the cytoplasmic tail, near the transmembrane region, is in a dark grey box. Cysteine residues (¤), potential N-glycosylation sites (#) and potential cytoplasmic-tail phosphorylation sites (+) are marked at the top of the sequences. Seven independent clones were sequenced in both directions. Sequence data have been deposited at GenBank under accession nos. DQ013285 and DQ013284 (shown here). Both sequences differ by a glutamine deletion in position 744 in sequence DQ013284.

The PoCD11a cDNA sequence contains an ORF of 3519 bp [GenBank:DQ013285] or 3522 bp [GenBank:DQ013284] that codes for 1172 or 1173 aa followed by ~500 bp that constitute the 3'-UTR (Fig. 1). The 1173 aa mature PoCD11a contains a 23-residue putative leader peptide (residues 1–23), an extracellular domain of 1064 residues (24–1087), a single hydrophobic transmembrane region of 24 residues (1088–1111) and a short cytoplasmic tail of 62 residues (1112–1173) (Fig. 1). Six N-linked putative glycosylation sites (Asn-Xaa-Ser/Thr) are found in the extracellular domain (Fig. 1). The PoCD11a possesses 22 cysteine residues, among which one is located into the cytoplasmic tail (Fig. 1). A subset of integrin α chains (α_1_, α_2_, α_10_, α_11_, α_D_, α_E_, α_L_, α_M _and α_X_), including CD11a, contain an I-domain (for Inserted domain and also called α_L_I-domain or _L_A-domain) that is homologous to the family of von Willebrand Factor (vWF) A-type domains and to cartilage matrix protein [1,18]. The I-domain has been associated with ligand binding. Its three-dimensional structure consists of a five-stranded parallel β-sheet core surrounded on both faces by seven α-helices. A short antiparallel strand occurs on one edge of this sheet [19]. The I-domain (148–331) contains a metal ion-dependent adhesion site (MIDAS) (residues 160–164, 229, 262) [19] (Fig. 1). The I-domain crystallisation has demonstrated that a "closed" (low affinity) and an "open" (high affinity) forms exist, and that the major conformational changes during transition from the closed to open states include a rearrangement of the cation-coordinating residues in the MIDAS site, accompanied by a small inward movement of the α1 helix and a large downward shift of the mobile C-terminal α7 helix [20]. The extracellular domain of PoCD11a contains seven internal repeats that surround the I-domain [21]. The degree of identity is highest among the three COOH-terminal repeats (18–31%) and their central region (466–474, 528–536 and 588–596) is similar to the EF hand divalent cation-binding motifs of troponin C, parvalbumin and galactose binding protein [21] (Fig. 1). All the N-glycosylation sites and all but one cysteine residues are found outside the I-region and divalent cation binding motifs (Fig. 1), consistent with the hypothesis that these regions may undergo conformational changes important in ligand binding [21,22]. The cytoplasmic portion of PoCD11a contains three potential phosphorylation sites and also a conserved "GFFKR" basic sequence near the transmembrane region (Fig. 1). The integrins become constitutively active when this sequence is deleted, the GFFKR motif thus normally fixes the integrins in an inactive state [4,23].

Among the seven positive clones sequenced, two presented a supplementary "cag" codon (2230–2232) that codes for a glutamine (Gln, Q) in position 744 [GenBank:DQ013284]. This addition is located in the extracellular domain of PoCD11a, outside of the I-domain and divalent cation-binding motifs and, according to the GORIV bioinformatic program, increased the length of an α-helix. The Gln-744 addition was also observed in the human [GenBank:NM_002209, GenBank:AY892236] and ovine [24] CD11a cDNAs. The Gln addition could thus have a biological importance for the mature CD11a. Studies of genomic sequences will permit to know if this addition represents two alleles or if it is generated by an alternative splicing.

### Comparison among species

**Figure 2:**
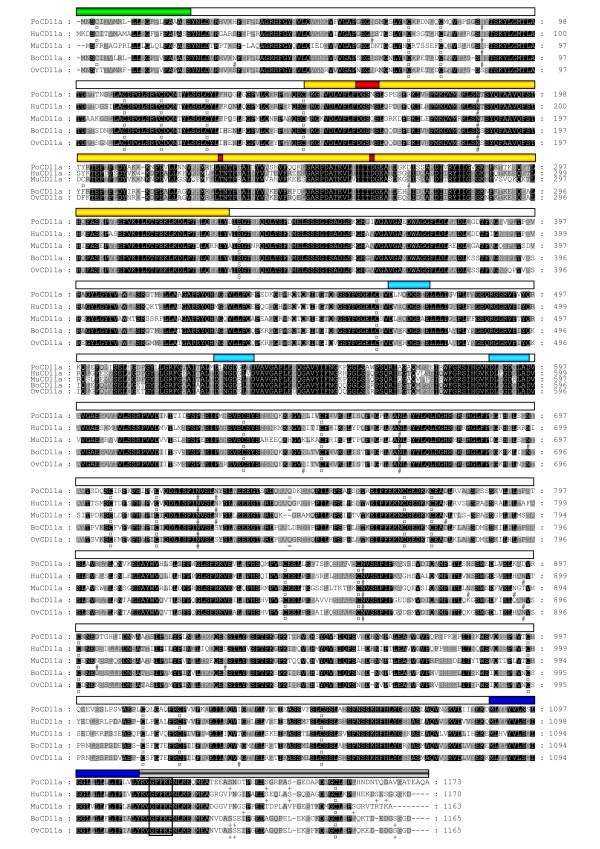
**Comparison of the porcine (Po-), human (Hu-), murine (Mu-), bovine (Bo-) and ovine (Ov-) α subunits amino acids sequences**. Black column with white letter, dark gray column with white letter and light gray column with black letter represent identity among 5, 4 and 3 species, respectively. Cysteine residues (¤), potential N-glycosylation sites (#) and potential cytoplasmic-tail phosphorylation sites (+) are marked at the bottom of the sequences. The important Glu-333 residue (E) and the Gln-744 residue (Q) are respectively identified by ($) and (=). The stripes above the sequences represent the deduced different constitutive parts of the protein: signal peptide (), extracellular domain (), transmembrane region (), cytoplasmic tail (),αI-domain () and its metal-ion dependent adhesion site (), and the central divalent cation-binding motifs of the three COOH-terminal repeats (). The highly conserved "GFFKR" motif of the cytoplasmic tail is framed for the different species.

Overall, the general organization of porcine, human [21], murine [25], bovine [26] and ovine [24] CD11a proteins is quite similar (Fig. 2). Comparison between mature PoCD11a sequence and its human, murine, bovine and ovine counterparts shows overall 77%, 69%, 78% and 77% identity, respectively, with the highest identity for the MIDAS, the cation binding motifs and the transmembrane region and the lowest identity for the cytoplasmic tail (Table 1). The high conservation of the MIDAS and the putative cation binding motifs is consistent with an involvement of these regions in the functional activity of LFA-1 α subunit, as suggested by the requirement of Mg^2+ ^and Ca^2+ ^for CD11a/CD18-dependent cellular interactions [22] or binding to purified ICAM-1 [27,28]. The transmembrane region shows also a high degree of conservation that could be explained by (i) physicochemical, and (ii) functional constraints. Indeed, (i) residues lying in the membrane have to possess a hydrophobic character to warrant liposolubility, which is confirmed by the presence of many leucine residues (fig. 2) and (ii) bi-directional integrin signalling (inside-out and outside-in) is accomplished by transmission of information across the plasma membrane [29]. By contrast, the low conservation of the COOH-terminal part of the cytoplasmic tail suggests that it is not required to guarantee adequate functioning of LFA-1. This is in agreement with the observation that truncation of the LFA-1 α subunit cytoplasmic domain has no effect on binding to ICAM-1, whereas binding is markedly diminished by β subunit cytoplasmic domain truncation [30]. However, the part near the transmembrane region of the cytoplasmic tail, containing the "GFFKR" sequence, is highly conserved (Table 1). This is consistent with the stabilizing role of this motif for the *alpha*/*beta *complex, possibly because of its direct involvement in heterodimer formation [23]. Residue Glu-333 that is located in the linker following the I domain and that is known to be critical for communication to the β_2 _I-like domain, rolling, integrin extension and activation by Mn^2+ ^of firm adhesion [31] is strictly conserved, too (fig. 2).

**Table 1:** Between-species percent identities of CD11a constitutive blocks. Po, Hu, Mu, Bo and Ov : porcine, human, murine, bovine and ovine CD11a, respectively ; MIDAS: metal-ion dependent adhesion site ; vs : versus.

**Block**	**Po vs. Hu (%)**	**Po vs. Mu (%)**	**Po vs. Bo (%)**	**Po vs.Ov (%)**
**Overall**	77	69	78	77
Putative signal peptide	56	45	86	78
Extracellular domain	77	70	77	78
I-domain	79	72	82	82
MIDAS	85	85	85	85
Putative cation binding motif 1	77	77	66	66
Putative cation binding motif 2	77	77	77	77
Putative cation binding motif 3	88	88	100	100
Transmembrane region	91	75	83	87
Cytoplasmic tail	55	47	55	53
"GFFKR" motif	100	100	100	100

Every cysteine residue in the mature porcine CD11a is present at the same location in human, murine, bovine and ovine CD11a, which is consistent with a role in maintaining the global structure of the protein. The mouse version distinguishes by an additional cysteine residue at position 199 within the extracellular portion. Of six potential Asn-glycosylation sites in porcine CD11a, the ones present at amino acids 186, 668, 724 and 860 are strictly conserved. Without predictable consequences on a functional point of view, one glycosylation site is only absent from porcine and murine CD11a (residue 897 of human sequence). The mouse sequence shows additional glycosylation sites at position 270 and 776. Furthermore, the porcine Asn-Xaa-Ser/Thr sites in position 87 and 728 are also found in human and murine homologues but not specially in the ruminant sequences, and the ovine sequence owns two supplementary sites at position 646 and 1000.

## Conclusion

This study reports for the first time the isolation and sequencing of the porcine LFA-1 α_L _subunit (CD11a) cDNA, and demonstrates that, despite some focal differences, it shares all the main characteristics of its known mammalian homologues. Along with the porcine CD18-encoding cDNA which is available [17], the sequence data provided here allow the successful cloning of PoCD11a, thus giving the first opportunity to express porcine LFA-1 *in vitro *as a tool to examine the specificities of inflammation in the porcine species.

## Methods

### RNA isolation

Total RNA from spleen of a freshly slaughtered pig (*Sus scrofa domestica*) was extracted with TRIzol (Invitrogen, USA) as described by the manufacturer.

### Amplification of cDNA ends

We used SMART RACE technology (Clontech Laboratories Inc., USA) to obtain porcine CD11a (PoCD11a) 5'- and 3'- ends and RT-PCR to amplify full-length PoCD11a CDS. For first strand cDNA synthesis, and according to the bovine CD11a sequence available [GenBank:AY267467], gene-specific primers were designed which were expected to give nonoverlapping ~1,5 kb rapid amplification of cDNA ends (RACE) products : a sense primer for the 3'-RACE PCR 5'-tgcaatgtragctctcccatcttc-3' (corresponding to nt 2575–2598) and an antisense primer for the 5'-RACE PCR 5'-aagatgtacacrgccccctgctcctcca-3' (nt 1628–1655). Reverse transcription and polymerase chain reactions (PCR) were carried out according to the instruction manual of the SMART RACE cDNA Amplification Kit. The 5'- and 3'-RACE products were gel-purified using the Qiaquick Gel Extraction Kit (Qiagen), TA-cloned into pCRII-TOPO (Invitrogen, USA) and seeded on kanamycin IPTG plates. Minipreps were obtained from colonies grown in 5 ml LB-Kan broth and the clones were sequenced on the ABI-3100 Genetic Analyzer using the Big Dye terminator chemistry (Applied Biosystems, USA).

### Molecular cloning of full-length cDNA

Total RNA from spleen cells was reverse transcribed using Improm II (Promega, USA). The full-length cDNA was then generated by long distance PCR using elongase amplification technology (Invitrogen, USA) with primers designed from the proximal part of 5'- and the distal part of 3'-RACE products: 5'-ggtatggtccctccagaagc-3' (forward) and 5'-tcaggcctgggcttcagtcg-3' (reverse). The following cycling parameters were applied: 5 min at 94°C, then 35 cycles including: (i) 30 s at 94°C, (ii) 45 s at 58°C, and (iii) 3 min 30 s at 68°C, followed by a final extension at 68°C for 10 min. Resulting PCR products were then processed for sequencing as aforementioned for the RACE products. The CD11a cDNA sequence was deduced from sequences obtained from seven independent clones. Sequence data have been deposited at GenBank under accession nos. DQ013285 and DQ013284.

### Bioinformatics

Primers design was performed with Netprimer [32] and Primer 3 [33]. Nucleotidic sequence and identity analyses were carried out using respectively Chromas v.2.21 [34] and BLAST programs [35]. Alignment of amino acids sequences was drawn by GeneDoc v.2.6.002 [36] following the blosum 62 matrix. SignalP v.2.0.b2 [37] and NetNGlyc v.1.0 [38] provided peptide signal and N-glycosylation sites prediction, respectively. The secondary structures were resolved by the GOR secondary structure prediction method version IV [39].

## List of abbreviations

aa, amino acid ; Bo, bovine; CD, cluster of differenciation ; CR, complement receptor ; DCBM, divalent-cation binding motif ; Hu, human; ICAM, intercellular adhesion molecule; kan, kanamycin ; LFA, lymphocyte function-associated antigen; MIDAS, metal-ion dependent adhesion site; Mu, murine; Ov, ovine ; Po, porcine ; RACE, rapid amplification of cDNA ends ; TCR, T-cell receptor.

## Authors' contributions

PVB carried out cloning, sequencing, sequence alignment and drafting of the manuscript. TF, LZ and AT participated in the design of the study and helped to structure analysis. DD conceived the study, and participated in its design and coordination and helped to draft the manuscript. All authors read and approved the final manuscript.

## References

[B1] Springer TA (1990). Adhesion receptors of the immune system. Nature.

[B2] Dib K (2000). *Beta *2 integrin signaling in leukocytes. Front Biosci.

[B3] Humphries MJ, Symonds EJ, Mould AP (2003). Mapping functional residues onto integrin crystal structures. Curr Opin Struct Biol.

[B4] Gahmberg CG, Valmu L, Fagerholm S, Kotovuori P, Ihanus E, Tian L, Pessa-Morikawa T (1998). Leukocyte integrins and inflammation. Cell Mol Life Sci.

[B5] Arnaout MA (1990). Leukocyte adhesion molecules deficiency: its structural basis, pathophysiology and implications for modulating the inflammatory response. Immunol Rev.

[B6] Trowald-Wigh G, Hakansson L, Johannisson A, Norrgren L, Hard af Segerstad C (1992). Leucocyte adhesion protein deficiency in Irish setter dogs. Vet Immunol Immunopathol.

[B7] Nagahata H, Kehrli ME, Murata H, Okada H, Noda H, Kociba GJ (1994). Neurtrophil function and pathologic findings in Holstein calves with leukocyte adhesion deficiency. Am J Vet Res.

[B8] Marlin SD, Springer TA (1987). Purified intercellular adhesion molecule-1 (ICAM-1) is a ligand for lymphocyte function-associated antigen 1 (LFA-1). Cell.

[B9] Simmons D, Makgoba MW, Seed B (1988). ICAM, an adhesion ligand of LFA-1, is homologous to the neural cell adhesion molecule NCAM. Nature.

[B10] Staunton DE, Dustin ML, Springer TA (1989). Functional cloning of ICAM-2, a cell adhesion ligand for LFA-1 homologous to ICAM-1. Nature.

[B11] Fawcett J, Holness CL, Needham LA, Turley H, Gatter KC, Mason DY, Simmons DL (1992). Molecular cloning of ICAM-3, a third ligand for LFA-1, constitutively expressed on resting leukocytes. Nature.

[B12] Ihanus E, Uotila L, Toivanen A, Stefanidakis M, Bailly P, Cartron JP, Gahmberg CG (2003). Characterization of ICAM-4 binding to the I domains of the CD11a/CD18 and CD11b/CD18 leukocyte integrins. Eur J Biochem.

[B13] Lally ET, Kieba IR, Sato A, Green CL, Rosenbloom J, Korostoff J, Wang JF, Shenker BJ, Ortlepp S, Robinson MK, Billings PC (1997). RTX toxins recognize a beta2 integrin on the surface of human target cells. J Biol Chem.

[B14] Zecchinon L, Fett T, Desmecht D (2005). How Mannheimia haemolytica defeats host defence through a kiss of death mechanism. Vet Res.

[B15] Bosse JT, Janson H, Sheehan BJ, Beddek AJ, Rycroft AN, Kroll JS, Langford PR (2002). Actinobacillus pleuropneumoniae: pathobiology and pathogenesis of infection. Microbes Infect.

[B16] Losinger WC (2005). Economic impacts of reduced pork production associated with the diagnosis of Actinobacillus pleuropneumoniae on grower/finisher swine operations in the United States. Prev Vet Med.

[B17] Lee J-K, Schook LB, Rutherford MS (1996). Molecular cloning and characterization of the porcine CD18 leukocyte adhesion molecule. Xenotransplantation.

[B18] Colombatti A, Bonaldo P (1991). The superfamily of proteins with von Willebrand factor type A-like domains: one theme common to components of extracellular matrix, hemostasis, cellular adhesion, and defense mechanisms. Blood.

[B19] Qu A, Leahy DJ (1995). Crystal structure of the I-domain from the CD11a/CD18 (LFA-1, alpha L beta 2) integrin. Proc Natl Acad Sci USA.

[B20] Mould AP, Humphries MJ (2004). Regulation of integrin function through conformational complexity: not simply a knee-jerk reaction?. Curr Opin Cell Biol.

[B21] Larson RS, Corbi AL, Berman L, Springer T (1989). Primary structure of the leukocyte function-associated molecule-1 alpha subunit: an integrin with an embedded domain defining a protein superfamily. J Cell Biol.

[B22] Rothlein R, Springer TA (1986). The requirement for lymphocyte function-associated antigen 1 in homotypic leukocyte adhesion stimulated by phorbol ester. J Exp Med.

[B23] Pardi R, Bossi G, Inverardi L, Rovida E, Bender JR (1995). Conserved regions in the cytoplasmic domains of the leukocyte integrin alpha L beta 2 are involved in endoplasmic reticulum retention, dimerization, and cytoskeletal association. J Immunol.

[B24] Fett T, Zecchinon L, Baise E, Desmecht D (2005). Cloning and characterization of the primary structure of the sheep lymphocyte function-associated antigen-1 alpha subunit. Mol Immunol.

[B25] Kaufmann Y, Tseng E, Springer TA (1991). Cloning of the murine lymphocyte function-associated molecule-1 alpha-subunit and its expression in COS cells. J Immunol.

[B26] Fett T, Zecchinon L, Baise E, Desmecht D (2004). The bovine (Bos taurus) CD11a-encoding cDNA: molecular cloning, characterisation and comparison with the human and murine glycoproteins. Gene.

[B27] Dustin ML, Springer TA (1989). T-cell receptor cross-linking transiently stimulates adhesiveness through LFA-1. Nature.

[B28] Vitte J, Pierres A, Benoliel AM, Bongrand P (2004). Direct quantification of the modulation of interaction between cell- or surface-bound LFA-1 and ICAM-1. Leukoc Biol.

[B29] Kim M, Carman CV, Springer TA (2003). Bidirectional transmembrane signaling by cytoplasmic domain separation in integrins. Science.

[B30] Hibbs ML, Xu H, Stacker SA, Springer TA (1991). Regulation of adhesion of ICAM-1 by the cytoplasmic domain of LFA-1 integrin beta subunit. Science.

[B31] Salas A, Shimaoka M, Kogan AN, Harwood C, von Andrian UH, Springer TA (2004). Rolling adhesion through an extended conformation of integrin alphaLbeta2 and relation to alpha I and beta I-like domain interaction. Immunity.

[B32] Netprimer. http://www.premierbiosoft.com/netprimer.

[B33] Rozen S, Skaletsky HJ (2000). Primer3 on the WWW for general users and for biologist programmers. Bioinformatics Methods and Protocols: Methods in Molecular Biology.

[B34] Chromas v.2.21. http://www.technelysium.com.au.

[B35] Altschul SF, Gish W, Miller W, Myers EW, Lipman DJ (1990). Basic local alignment search tool. J Mol Biol.

[B36] Nicholas B, Karl B, Nicholas P, Hugh B GeneDoc : a tool for editing and annotating multiple sequence alignments. http://www.psc.edu/biomed/genedoc.

[B37] Nielsen H, Engelbrecht J, Brunak S, von Heijne G (1997). Identification of prokaryotic and eukaryotic signal peptides and prediction of their cleavage sites. Protein Eng.

[B38] Jensen LJ, Gupta R, Blom N, Devos D, Tamames J, Kesmir C, Nielsen H, Staerfeldt HH, Rapacki K, Workman C, Andersen CA, Knudsen S, Krogh A, Valencia A, Brunak S (2002). Prediction of human protein function from post-translational modifications and localization features. J Mol Biol.

[B39] Garnier J, Gibrat JF, Robson B (1996). GOR method for predicting protein secondary structure from amino acid sequence. Methods Enzymol.

